# Use of renal risk drugs in a nation-wide Polish older adult population: an analysis of PolSenior database

**DOI:** 10.1186/s12877-019-1075-5

**Published:** 2019-03-05

**Authors:** Ewa Deskur-Śmielecka, Jerzy Chudek, Agnieszka Neumann-Podczaska, Małgorzata Mossakowska, Barbara Wizner, Katarzyna Wieczorowska-Tobis

**Affiliations:** 1 Chair and Department of Palliative Medicine, Poznan University of Medical Sciences, Hospicjum Palium, os. Rusa 55, 61-245 Poznan, Poland; 2 Palliative Medicine Unit, University Hospital of Lord’s Transfiguration, Poznan, Poland; 3 Department of Internal Medicine and Oncological Chemotherapy, Medical Faculty in Katowice, Medical University of Silesia, Katowice, Poland; 4 Chair of Geriatrics and Gerontology, Poznan University of Medical Sciences, Poznan, Poland; 5 International Institute of Molecular and Cell Biology in Warsaw, Warsaw, Poland; 6 Department of Internal Medicine and Gerontology, Jagiellonian University Medical College, Krakow, Poland

**Keywords:** Chronic kidney disease, Older adults, Inappropriate drug use, Dosage adjustment, Drug combinations

## Abstract

**Background:**

Numerous medications should be avoided, or require dose adjustment in subjects with impaired kidney function. We aimed to assess the prevalence of potentially inappropriate use of renal risk drugs in a nation-wide, community-dwelling Polish older adult population.

**Methods:**

We analysed regular intake of 38 medications that should be avoided, requiring dose modification, increase the risk of pre-renal kidney injury, or may cause potassium retention in subjects with moderately to severely impaired renal function in the PolSenior data base (*N* = 4514, mean age 76 ± 11 yrs). Kidney function was assessed with short Modification of Diet in Renal Disease formula estimated glomerular filtration rate (sMDRD) and Cockcroft-Gault creatinine clearance (CC).

**Results:**

There were 855 (19%) individuals with sMDRD < 60 ml/min/1.73m^2^, and 1734 (38%) with CC <  60 ml/min. Among drugs that should be avoided, spironolactone (20.4% of patients as classified by sMDRD and 17.5% by CC), non-steroidal anti-inflammatory drugs (13.4 and 11.3%), hydrochlorothiazide (11.1 and 11.0%), and metformin (6.9 and 8.2%) were most frequently used. The most frequently used drugs requiring dose modification were piracetam (13.9% by sMDRD, and 11.9% by CC), digoxin (8.3 and 8.8%), and gliclazide (6.8 and 5.9%). Classification of a drug use as ‘appropriate’ or ‘inappropriate’ was discordant depending on the method of kidney function assessment (sMDRD or CC) in up to 30%.

Subgroups with sMDRD < 60 ml/min/1.73m^2^ and with CC <  60 ml/min were taking ≥2 drugs increasing the risk of pre-renal kidney injury more frequently than individuals with better kidney function (46.6 vs. 23.1 and 33.0% vs. 24.4%, respectively).

There were 24.7% of individuals with sMDRD < 60 ml/min/1.73m^2^ and 18.0% with CC <  60 ml/min taking 2 or more drugs increasing serum potassium level. The proportion of subjects with hyperkalaemia increased with the number of such drugs.

**Conclusions:**

Use of drugs that should be avoided or require dose adjustment due to renal impairment, and potentially inappropriate drug combinations is a common problem in older adults in Poland. Assessment of kidney function with sMDRD may result in overlooking of requirements for dose adjustment formulated based on creatinine clearance.

**Trial registration:**

Not applicable.

## Background

Chronic kidney disease (CKD) is a common entity among older adult subjects. After the age of 40, kidney function assessed with creatinine clearance decreases by 8–9 ml/min with each decade of life [1, 2]. This decrease results from a normal biological process of aging and superimposing diseases, such as diabetes and hypertension [3]. The prevalence of CKD in the general population is estimated at approximately 11% [4–6] and increases with age reaching 45% in subjects aged 80 years or more [7, 8].

Numerous medications commonly used in the older adults should be avoided in subjects with impaired kidney function because of their nephrotoxic properties, e.g., non-steroidal anti-inflammatory drugs (NSAIDs), angiotensin-converting enzyme inhibitors (ACEI), methotrexate, loop and thiazide diuretics, ranitidine, some antibiotics [8]. Other drugs, e.g., digoxin, gliclazide, and atenolol are excreted by the kidney and require dose adjustment in subjects with impaired kidney function [9]. The process of drug selection and dose-adjustment is particularly challenging in the older population, because of common comorbidity and associated polypharmacy [10]. Incidences of adverse drug reactions in patients with renal impairment are higher than in subjects with normal kidney function [11]. In the GIFA study (Gruppo Italiano di Farmacovigilanza nell’Anziano; the Italian Group of Farmacoepidemiology in the Elderly) low estimated glomerular filtration rate (eGFR) or creatinine clearance were significantly more prevalent in hospitalized elderly patients with adverse reactions caused by hydrosoluble drugs, esp. diuretics, digoxin, angiotensin-converting enzyme inhibitors (ACEI), and antihyperglycemic agents [12]. Adverse drug reactions often result from errors in prescribing or the lack of dose adjustment [13]. Despite the recognized importance of inappropriate drug prescriptions in older adults with CKD, the appropriate choice of medication and dosage adjustment are often neglected in daily clinical practice. Partially it is caused by utilization of eGFR provided by laboratories along with serum concentration of creatinine, calculated according to the short Modification of Diet in Renal Disease (sMDRD), or the Chronic Kidney Disease Epidemiology Collaboration (CKD-EPI) formulas, and skipping calculation of Cockcroft-Gault creatinine clearance (CC) used in drug registration process and required for dose adjustment.

Several studies have shown that the prevalence of inappropriate prescriptions in hospitalized older adults with renal impairment varies from 9 to 67% [14–19]. Regardless of the fact that in older adults with CKD most drugs are prescribed in an outpatient setting, few studies have investigated the problem of inappropriate drug use in this population. In a recent systematic review, Dorks et al. [20] have identified the lack of dose adjustment for renal function as a common problem, ranging from 6 to 43% of inappropriate prescriptions in nursing homes and 1–37% in outpatient settings. Of note, only four studies included in the review [21–24] were large, population-based analysis. The authors of the review emphasized the importance of further investigation in this field.

## Aim

We analyzed the PolSenior database to assess the prevalence of regular intake of renal risk drugs (defined as medications that should be avoided, or that require dose adjustment in subjects with impaired kidney function) in the community-dwelling Polish older adult population. Additionally, we assessed the prevalence of potentially inappropriate drug combinations in such individuals.

## Methods

We retrospectively analyzed data from the PolSenior study, a multidisciplinary research project, conducted in 2007–2011, to assess the medical, psychological, social and economic aspects of aging in Poland. Participants were selected through a multi-stage draw, planned to obtain a sample representative for Polish old population. A detailed description of the study design has already been presented elsewhere [25].

Renal function was assessed with glomerular filtration rate calculated with the short Modification of Diet in Renal Disease formula (sMDRD) [26] as serum creatinine concentrations were measured using the Jaffe method (modular PPE, Roche Diagnostics GmbH, Mannheim, Germany), and creatinine clearance (CC) calculated with the Cockcroft-Gault formula [27].

We analyzed regular (≥ 3 times per week) intake of medications that should be avoided, or requiring dose modification when used in subjects with moderately (eGFR < 50–60 ml/min) to severely (eGFR < 25–30 ml/min) impaired renal function. The selection of drugs chosen for analysis was based on guidelines from the American Geriatrics Society [28] and consensus guidelines for oral dosing of primarily renally cleared medications in older adults by Hanlon et al. [29]. Additionally, several medications commonly used in older patients were analyzed based on information given by Ashley et al. [9]. Only drugs registered in Poland were assessed. Finally, 38 drugs were selected for analysis (Tab. 1). For each drug, the number and percentage of subjects taking it in the total study population were calculated. Next, the number and percentage of given drug users among individuals with kidney function below and above the level indicated in the guidelines (Tab. 1) were calculated and classified as potentially inappropriate use and renally appropriate use. Calculations were performed for both creatinine clearance calculated with the Cockcroft-Gault formula and sMDRD-derived eGFR. In the same manner, we analyzed potentially inappropriate combinations of drugs:

**Table 1:** List of drugs chosen for analysis and prescription recommendations in patients with impaired renal function

Name of drug	Recommendations	Reference
Gastrointestinal drugs		
Metoclopramide	Avoid < 40 ml/min	28
Famotidine	Reduce dose < 50 ml/min	27,28
Ranitidine	Reduce dose < 50 ml/min	27,28
Antidiabetics		
Metformin	Avoid < 60 ml/min	28
Acarbose	Avoid < 25 ml/min	9
Gliclazide	Reduce dose < 50 ml/min	9
Antithrombotic agents		
Dabigatran	Avoid < 30 ml/min	27
Rivaroxaban	Reduce dose < 50 ml/min	27
Rivaroxaban	Avoid < 30 ml/min	27
Enoxaparin	Reduce dose < 30 ml/min	27
Cardiovascular drugs		
Digoxin	Reduce dose < 50 ml/min	9
Sotalol	Avoid < 40 ml/min	28
Atenolol	Reduce dose < 35 ml/min	28
Amiloride	Avoid < 30 ml/min	27
Spironolactone	Avoid < 30 ml/min	27
Hydrochlorothiazide	Avoid < 30 ml/min	27
Rosuvastatin	Reduce dose < 60 ml/min	9
Fenofibrate	Reduce dose < 60 ml/min	9
Antianalgesics		
Tramadol	Reduce dose < 30 ml/min	27
Morphine	Reduce dose < 50 ml/min	9
Oxycodone	Reduce dose < 50 ml/min	9
NSAIDs	Avoid < 50 ml/min	9
Antidepressants		
Duloxetine	Avoid < 30 ml/min	27
Bupropion	Reduce dose < 50 ml/min	9
Venlafaxine	Reduce dose < 30 ml/min	9
Anti-epileptic drugs		
Gabapentin	Reduce dose < 60 ml/min	27,28
Pregabalin	Reduce dose < 60 ml/min	27
Levetiracetam	Reduce dose < 80 ml/min	9,27
Topiramate	Reduce dose < 70 ml/min	27
Other drugs		
Piracetam	Reduce dose < 50 ml/min	9
Risperidone	Reduce dose < 50 ml/min	9
Sulpiride	Reduce dose < 50 ml/min	9
Cetirizine	Reduce dose < 30 ml/min	28
Fexofenadine	Reduce dose < 80 ml/min	28
Tizanidine	Reduce dose < 25 ml/min	9
Colchicine	Reduce dose < 30 ml/min	27
Alendronic acid	Avoid < 35 ml/min	9
Methotrexate	Reduce dose < 50 ml/min	9
Memantine	Reduce dose < 30 ml/min	9,28

*NSAIDs* Nonsteroidal anti-inflammatory drugs

1) concomitant use of drugs that increase the risk of pre-renal kidney injury [combination of nonsteroidal anti-inflammatory drugs (NSAIDs), diuretics, ACEI, and angiotensin II receptor blockers (ARB)],

2) combination of drugs resulting in potassium retention (ACEI, ARB, aldosterone antagonists, potassium-sparing diuretics, and potassium-containing agents).

## Statistical analysis

Statistical analysis was performed with StatSoft Statistica v12. Data are shown as mean ± standard deviation or numbers (%). Percentage of particular drug users among subjects with eGFR and CC below and above the level indicated in the recommendations was compared with the chi^2^ test with Yates correction (if appropriate). For potentially inappropriate drug combinations, we performed comparisons between patients with eGFR or CC below and above 60 ml/min/1.73m^2^ or 60 ml/min, respectively. *P* value < 0.05 was considered significant.

## Results

Data concerning 5695 subjects were included in the PolSenior database. After exclusion of 1181 subjects in whom blood samples were not drown for serum creatinine assessment or anthropometric data were missing, data on 4514 (79.3%) subjects [2177 (48.2%) women] were included in the analysis. The mean age was 76 ± 11 yrs.

Among study group, 855 participants had CKD stage 3–5 (staging according to the National Kidney Foundation classification is presented in Table 2) [30]. The mean sMDRD was 78.3 ± 22.6 ml/min/1.73m^2^ and CC was 79.5 ± 29.1 ml/min.

**Table 2:** Renal function staged using the National Kidney Foundation classification (total number of subjects = 4514)

CKD stage	sMDRD (ml/min/1.73m^2^)	CC (ml/min)
1 (eGFR ≥90); n (%)	1206 (26.7)	1048 (23.2)
2 (eGFR 60–89); n (%)	2453 (54.3)	1732 (38.4)
3 (eGFR 30–59); n (%)	800 (17.7)	1533 (34.0)
4 (eGFR 15–29); n (%)	50 (1.1)	187 (4.1)
5 (eGFR < 15); n (%)	5 (0.1)	14 (0.3)

*sMDRD* short Modification of Diet in Renal Disease study equation; *CC* Creatinine clearance calculated with Cockcroft-Gault; *CKD* Chronic kidney disease

Of 4514 individuals in the study group, 1916 (42.4%) subjects were regularly taking at least one of 38 analyzed drugs. Among individuals with sMDRD and CC below 60 ml/min/1.73m^2^ and ml/min, the prevalence of potentially inappropriate drug use was 41.2 and 36.8%, respectively. In subjects with sMDRD and CC below 30 ml/min/1.73m^2^ and ml/min, the prevalence was 58.2 and 57.7%.

Detailed data concerning the use of renal risk drugs in subjects with the renal function below and above the level indicated in the recommendations (potentially inappropriate use and renally appropriate use) is presented in Table 3. Twenty-one drugs were taken only occasionally (in less than 20 individuals), and they are not shown in Table 3 for clarity reasons (bupropion, colchicine, dabigatran, duloxetine, enoxaparin, famotidine, fexofenadine, gabapentin, levetiracetam, memantine, methotrexate, morphine, oxycodone, pregabalin, risperidone, rivaroxaban, rosuvastatin, sulpiride, tizanidine, topiramate, and venlafaxine).

**Table 3:** Participants taking renal risk drugs (requiring dose adjustment or drug that should be avoided; total number of subjects 4514)

Name of drug	Total population; n (%)	CC			sMDRD			Appropriate by sMDRD but inappropriate by CC (n; % of all patients taking particular drug)
		Potentially inappropriate use; n (% of subjects with CC below level indicated in recommendations)	Renally appropriate use; n (% of subjects with CC above level indicated in recommendations)	Potentially inappropriate use; % of all patients taking particular drug	Potentially inappropriate use; n (% of subjects with sMDRD below level indicated in recommendations)	Renally appropriate use; n (% of subjects with sMDRD above level indicated in recommendations)	Potentially inappropriate use; % of all patients taking particular drug	
Drugs that should be avoided								
Metoclopramide	20 (0.44)	4 (0.74)	16 (0.40)	20.00%	1 (0.62)	19 (0.44)	5.00%	3 (15.0)
Metformin	292 (6.47)	64 (3.69)*	228 (8.20)	21.92%	59 (6.90)	233 (6.37)	20.20%	24 (8.2)
Acarbose	39 (0.86)	2 (2.00)	37 (0.84)	5.13%	1 (4.35)	38 (0.85)	2.56%	1 (2.6)
Sotalol	63 (1.40)	4 (0.74)	59 (1.49)	6.35%	1 (0.62)	62 (1.42)	1.59%	3 (4.8)
Amiloride	126 (2.79)	13 (6.44)*	113 (2.62)	10.32%	4 (4.26)	122 (2.76)	3.17%	12 (9.5)
Spironolactone	330 (7.31)	35 (17.50)*	295 (6.84)	10.61%	11 (20.37)*	319 (7.15)	3.33%	25 (7.6)
Hydrochlorothiazide	265 (5.87)	22 (11.00)*	243 (5.64)	8.30%	6 (11.11)	259 (5.81)	2.26%	18 (6.8)
NSAIDs^&^	525 (11.63)	122 (11.31)	403 (11.74)	23.24%	55 (13.41)	470 (11.45)	10.48%	73 (13.9)
Meloxicam and nimesulide	108 (2.39)	25 (2.32)	83 (2.42)	23.15%	11 (2.68)	97 (2.36)	10.18%	15 (13.9)
Alendronic acid	20 (0.44)	2 (0.59)	18 (0.43)	10.00%	0	20 (0.45)	0%	2 (10.00)
Drugs requiring dose adjustment								
Ranitidine	100 (2.22)	42 (3.89)*	58 (1.69)	42.00%	15 (3.66)*	85 (2.07)	15.00%	28 (28.0)
Gliclazide	197 (4.36)	56 (5.19)	141 (4.11)	28.43%	28 (6.83)*	169 (4.12)	14.21%	32 (16.2)
Digoxin	204 (4.52)	95 (8.80)*	109 (3.18)	46.57%	34 (8.29)*	170 (4.14)	16.67%	63 (30.9)
Atenolol	56 (1.24)	5 (1.48)	51 (1.22)	8.93%	4 (4.12)*	52 (1.18)	7.14%	1 (1.8)
Fenofibrate	61 (1.35)	24 (1.38)	37 (1.33)	39.34%	24 (2.81)*	37 (1.01)	39.34%	4 (6.6)
Tramadol	81 (1.79)	3 (1.50)	78 (1.81)	3.70%	0	81 (1.82)	0%	2 (2.5)
Piracetam	342 (7.58)	128 (11.86)*	214 (6.23)	37.43%	57 (13.90)*	285 (6.94)	16.67%	77 (22.5)
Cetirizine	38 (0.84)	0	38 (0.88)	0%	0	38 (0.85)	0%	0

^&^excluding meloxicam, nimesulide, and low-dose aspirine

*p < 0.05 vs. renally appropriate use

*sMDRD* short Modification of Diet in Renal Disease study equation; *CC* Creatinine clearance calculated with Cockcroft-Gault equation

Among medications that should be avoided, spironolactone (prevalence of potentially inappropriate use 17.5% by CC, and 20.4% by sMDRD), NSAIDs (11.3 and 13.4%), hydrochlorothiazide (11.0 and 11.1%), metformin (8.2 and 6.9%), and amiloride (6.4 and 4.3%) were most frequently used. The most frequently used drugs requiring dose modification at given level of renal impairment were piracetam (prevalence of potentially inappropriate use 11.9% by CC, and 13.9% by sMDRD), digoxin (8.8 and 8.3%), gliclazide (5.9 and 6.8%), and ranitidine (3.9 and 3.7%).

We identified individuals taking a combination of two or more drugs increasing the risk of pre-renal impairment of kidney function (diuretics + NSAIDs + ACEI or ARB) (Table 4). In the total population, 1251 (27.7%) of subjects were taking 2–5 of such drugs. Intake of two or more drugs was more frequent in patients with CKD stage 3–5 as compared with subjects with better kidney function, despite the method of kidney function evaluation (CC: 33.0% of patients vs. 24.4%, *p* < 0.05; sMDRD: 46.6 vs. 23.1%, p < 0.05). Combinations of ACEI/ARB and diuretics were more prevalent in subjects with CC <  60 ml/min or sMDRD < 60 ml/min/1.73m^2^ as compared to individuals with better kidney function. Combinations of NSAIDS and diuretics, as well as triple combinations, were more frequently used by subjects with sMDRD < 60 ml/min/1.73m^2^ as compared to those with higher sMDRD.

**Table 4:** Subjects taking drugs increasing risk of pre-renal impairment of kidney function (nonsteroidal anti-inflammatory drugs + angiotensin-converting enzyme inhibitors/angiotensin II receptor blockers + diuretics)

Drug combinations	Total population (*n* = 4514)	CC		sMDRD	
		< 60 ml/min (*n* = 1734)	≥ 60 ml/min (*n* = 2780)	< 60 ml/min/1.73m^2^ (*n* = 855)	≥ 60 ml/min/1.73m^2^ (*n* = 3659)
ACEI/ARB + diuretic; n (%)	840 (16.61)	391 (22.55)*	449 (16.15)	278 (32.51)^#^	562 (15.36)
ACEI/ARB + NSAID; n (%)	225 (4.56)	86 (4.59)	139 (5.00)	50 (5.85)	175 (4.78)
Diuretic + NSAID; n (%)	163 (3.61)	71 (4.09)	92 (3.31)	54 (6.32)^#^	109 (2.98)
ACEI/ARB + diuretic + NSAID; n (%)	98 (2.17)	39 (2.25)	59 (2.11)	31 (3.63)^#^	67 (1.83)

*p < 0.05 vs. patients with CC ≥ 60 ml/min

^#^*p* < 0.05 vs. patients with sMDRD ≥60 ml/min/1.73m^2^

*ACEI* Angiotensin-converting enzyme inhibitor; sMDRD = short Modification of Diet in Renal Disease study equation; ARB = angiotensin II receptor blocker; CC = creatinine clearance; NSAID = nonsteroidal anti-inflammatory drug

We also identified individuals taking a combination of 2 or more drugs that may result in hyperkalaemia (ACEI, ARB, aldosterone antagonist, potassium-sparing diuretics, potassium-containing agents) (Table 5). Regardless of the method of kidney function assessment, subjects with CKD stage 3–5 were taking ≥2 drugs increasing potassium level more frequently than subjects with better kidney function (sMDRD: 18.1 vs. 10.9%, *p* < 0.05; CC: 24.7 vs. 11.0%, p < 0.05). The prevalence of hyperkalaemia in subjects taking 1, 2 or 3 drugs was higher in subjects with CC or sMDRD < 60 ml/min/1.73m^2^ as compared to those with better kidney function (Table 6). Chi-square test for trend showed that the proportion of subjects with hyperkalaemia increased significantly with the number of drugs in three of four analyzed subgroups: subjects with CC <  60 ml/min, individuals with CC ≥ 60 ml/min, and a subgroup with sMDRD < 60 ml/min/1.73m^2^.

**Table 5:** Concomitant use of drugs increasing serum potassium level (angiotensin-converting enzyme inhibitors + angiotensin II receptor blockers + aldosterone antagonists + potassium-sparing diuretics + potassium-containing agents)

No. of drugs	Total population (*n* = 4514)	CC		sMDRD	
		< 60 ml/min (*n* = 1734)	≥ 60 ml/min (*n* = 2780)	< 60 ml/min/1.73m^2^ (*n* = 855)	≥ 60 ml/min/1.73m^2^ (*n* = 3659)
1 drug; n (%) of patients	1621 (35.91)	669 (38.58)	952 (34.24)	391 (45.73)	1230 (33.62)
2 drugs; n (%) of patients	506 (11.21)	258 (14.88)	248 (8.92)	180 (21.05)	326 (8.91)
3 drugs; n (%) of patients	105 (2.33)	54 (3.11)	51 (1.83)	30 (3.51)	75 (2.05)
4 drugs; n (%) of patients	4 (0.09)	1 (0.06)	3 (0.11)	1 (0.12)	3 (0.08)

sMDRD = short Modification of Diet in Renal Disease study equation; *CC* Creatinine clearance calculated with Cockcroft-Gault formula

**Table 6:** Prevalence of hyperkalaemia among subjects taking 1–4 drugs increasing serum potassium level (angiotensin-converting enzyme inhibitors + angiotensin II receptor blockers + aldosterone antagonists + potassium-sparing diuretics + potassium-containing agents)

No. of drugs	CC		sMDRD	
	< 60 ml/min	≥ 60 ml/min	< 60 ml/min/1.73m^2^	≥ 60 ml/min/1.73m^2^
1; n (%) of subjects with hyperkalaemia	102/669 (15.26)* ^&^	73/952 (7.67) ^&^	73/391 (18.67)^# &^	102/1230 (8.29)
2; n (%) of subjects with hyperkalaemia	57/258 (22.09)*	22/248 (8.87)	52/180 (28.89)^#^	27/326 (8.28)
3; n (%) of subjects with hyperkalaemia	14/54 (25.93)	13/51 (25.49)	14/30 (46.67)^#^	13/75 (17.33)
4; n (%) of subjects with hyperkalaemia	1/1 (100)	0/33 (0)	1/1 (100)	0/3 (0)

*p < 0.05 vs. patients with CC ≥ 60 ml/min

^#^*p* < 0.05 vs. patients with sMDRD ≥60 ml/min/1.73m^2^

^&^*p* < 0.05 for chi-square test for trend for number of drugs

*sMDRD* short Modification of Diet in Renal Disease study equation; *CC* Creatinine clearance calculated with Cockcroft-Gault equation

The prevalence of potentially inappropriate use of some drugs and drug combinations varied depending on the method of assessment of kidney function (Table 3-6). We identified subjects in whom sMDRD values were higher than indicated in the recommendations for a particular drug (renally appropriate use), but CC values were below this threshold (potentially inappropriate use; Table 3, last column). Discordant evaluation of ‘appropriateness’ of use ranged from 0 to 30%, and was highest for digoxin (30.9%), ranitidine (28.0%), and piracetam (22.5%).

## Discussion

In this analysis of the national, population-based PolSenior study, we assessed the use of renal risk drugs, i. e. drugs that should be avoided and medications requiring dose reduction in subjects with impaired renal function. We found that approximately 40% of subjects with CKD stage 3, and nearly 60% of individuals with CKD stage 4 and 5 were taking at least one medication that was contraindicated given their level of renal function, or for which there were renal dosing recommendations. The results of previous reports on the use of renal risk drugs in subjects with CKD vary widely, from 13.3% in the Three-City population-based study [21], 30–53% in large cohort of outpatient older adult subjects [22], up to 62% in a series of hospitalized patients [18] and 80.5% in a small Turkish study [31]. Comparison between results of these studies, as well as with the results of studies revised by Dorks et al. [20] should be made with caution because of various drug sets analyzed in each report, and different definitions of potentially inappropriate drug use in CKD patients. Although we have not analyzed if doses of drugs requiring modification actually exceeded the maximum recommended daily dose in patients with given level of kidney impairment, the high percentage of subjects with CKD stage 3–5 taking renal risk drugs indicates increased the risk of drug-related problems [18] and all-cause mortality [21].

The most frequently used drugs that should be avoided in patients with renal impairment were diuretics (spironolactone, hydrochlorothiazide, and amiloride), NSAIDs, and metformin. The most frequently used medications requiring dose adjustment were piracetam, digoxin, gliclazide and ranitidine (Table 3). While NSAIDs, metformin, diuretics, and ranitidine are frequently reported as being inappropriately prescribed in patients with renal impairment [20], high proportions of subjects taking digoxin and piracetam are surprising. The possible explanation is that neither digoxin nor piracetam is listed by Beers criteria [28] or consensus guidelines by Hanlon et al. [29]. Digoxin was excluded from some studies, as an assessment of serum concentrations and therapeutic response is important for the evaluation of ‘appropriateness’ of its use [16]. We included these drugs in our analysis based on their frequent use in the total PolSenior population and well-established recommendations for dose adjustment in CKD [9]. It should be emphasized that indications for their use have been limited since the time the PolSenior study was conducted, and thus the prevalence of inappropriate use of these drugs presumably should have diminished. For metformin, we used the restrictive recommendations to avoid its use if CC was below 60 ml/min [29]. Although such a threshold can be still found in product characteristics of metformin, some recent guidelines [32–34] recommend cautious continuation of its use in patients with CC above 30 ml/min. Such a change in recommendations would greatly influence the prevalence of potentially inappropriate prescriptions.

Comparison of prevalence of renal risk drug intake in subjects with kidney function below and above the level indicated in recommendations revealed that metformin was the only drug that was used less frequently in subjects with renal contraindications (Tab. 3). The prevalence of intake of the remaining drugs with renal recommendations did not differ between these groups of patients or was even higher in subjects with kidney function below the recommended level (esp. ranitidine, digoxin, spironolactone, and piracetam). The percent of subjects in whom the use of a renal risk drug was potentially inappropriate among all subjects taking this particular medication ranged from 0 to 46%, and was highest for digoxin, piracetam, fenofibrate, and ranitidine. These data should be interpreted with caution, as CKD is associated with numerous chronic conditions, including hypertension, congestive heart failure, atrial fibrillation, and diabetes [35–38], which may explain why subjects with CKD stage 3–5 frequently received diuretics, digoxin, metformin, or piracetam – a nootropic drug commonly prescribed for cognitive impairment and dementia despite its unproven efficacy [39]. Nevertheless, the widespread use of medications with renal recommendations in PolSenior subjects with impaired kidney function suggests that the prescribers’ adherence to these recommendations was poor. In addition, it may be caused by the lack of CC calculation and taking into consideration only eGFR provided by laboratories, when prescribing drugs.

It has been recognized that concurrent use of ACEI/ARB and diuretics with NSAIDs is associated with increased risk of renal adverse effects, particularly in the older adults [40]. Each of these drugs may cause pre-renal injury via different mechanisms: diuretics may cause hypovolaemia and reduce plasma flow, ACEI/ARB lead to efferent arteriolar vasodilation and reduce glomerular filtration rate, and NSAIDs inhibit prostaglandin-induced afferent arteriolar vasodilatation [40]. Patients taking 2 or 3 drugs from the groups above were found to have elevated creatinine levels [41], and the use of triple therapy (but not double therapy) was associated with 31% higher risk of acute renal failure [42]. Therefore, such combinations should be avoided, especially in the older adults. The prevalence of triple therapy in the PolSenior population was rather low, as compared to that reported by Loboz and Shenfield (2.2 vs. 6.3%), and the prevalence of double therapy was somewhat higher (24.8 vs. 19.9%) [41]. Importantly, the percentage of subjects receiving such combinations among patients with CKD stage ≥3 was similar to, or higher than in individuals with better kidney function (Table 4).

Hyperkalaemia is a common drug-related problem, especially in the older adults, in patients with moderate to severe CKD, and those taking combinations of drugs known to increase serum potassium levels [43]. Concurrent use of aldosterone antagonists and potassium conserving drugs without monitoring of serum potassium has been included in the modified STOPP criteria [44]. Over 13% of subjects in the PolSenior population was taking concurrently 2–3 drugs increasing serum potassium, and the prevalence of concomitant use of such drugs was higher in individuals with CKD stage ≥3 (Table 5). The prevalence of hyperkalaemia increased with the number of drugs taken, and was higher in subjects with moderate to severe CKD (reaching 62% in individuals with sMDRD < 60 ml/min/1.73m^2^ receiving 3 drugs). These findings indicate that potentially inappropriate combinations of drugs are prescribed without taking into consideration renal risk, and/or that renal function and serum potassium are not adequately monitored while using such combined therapy.

The prevalence of use of renal risk drugs and potentially inappropriate drug combinations differed depending on the method of assessment of kidney function (CC or sMDRD; Tables 3-6). For some drugs with the recommendation at the sMDRD level of 50–60 ml/min/1.73m^2^, e.g., piracetam, digoxin, or ranitidine, in 20 to 30% of patients their use was rated as ‘renally appropriate’ if kidney function was assessed with sMDRD-derived eGFR, but ‘potentially inappropriate’ by CC (Table 3). These differences stem from a noticeable disagreement between estimation of renal function with different formulas and only moderate agreement in CKD staging based on Cockcroft-Gault and sMDRD equations [45]. While kidney function in clinical practice is usually assessed with sMDRD or CKD-EPI glomerular filtration rate estimates, most manufacturers’ recommendations on drug dosage adjusting were developed based on CC calculated with the Cockcroft-Gault equation. Greater discrepancies between sMDRD and CC were observed in women, disabled (activities in daily living ≤4 pts), and subjects aged 80 years or older [46]. The discrepancies in CKD staging based on different formulas have been recognized to result in inappropriate dosing of renally excreted drugs [47–49].

## Limitations

There are several limitations to our analysis. First, the data from the PolSenior study did not comprise information about prescribers. Some medications could be prescribed by specialists against official recommendations for special conditions, e.g. spironolactone in patients with CKD and congestive heart failure. In such situations use of drugs with renal recommendations requires special attention and monitoring, but should not be classified as a treatment error. Therefore, we may only discuss the frequency of use of drugs with renal recommendations, or “potentially inappropriate” drug use, and the actual risk associated with inappropriate use may be lower. Some other drugs that should not be used in persons with impaired kidney function, especially some NSAIDs, are available over the counter, and may be used by patients without, or even against, their doctor’s recommendations. Second, the PolSenior study was performed in the years 2007–2011. The recommendations for many drugs use, e.g., metformin have changed since that time. Other medications, such as rivaroxaban, dabigatran, pregabalin, levetiracetam, or memantine, were not available in Poland at that time, or they were very expensive, and not used in everyday practice. Drugs with renal recommendations which were not used on a regular basis, especially antibiotics, were not included in our analysis. Next, for the reason of methodology issues, we did not analyze the doses of drugs with renal recommendations. Thus, the doses of renally cleared drugs might have been actually adjusted to patients’ renal function. However, this limitation does not refer to drugs that should be avoided in patients with a certain level of renal impairment. Finally, the list of medications chosen for analysis, and levels of renal impairment, may be questioned. The conflicting recommendations for dosing of renally cleared drugs from various sources have been recognized [50]. The lack of clear, handy guidelines for drug dosing in older patients with renal impairment may be a major obstacle to reduce the prevalence of inappropriate prescriptions.

## Conclusions

Potentially inappropriate use of drugs, that is the use of drugs that should be avoided in patients with renal impairment, medications requiring dose adjustment, and potentially inappropriate combinations of drugs (concomitant use of drugs increasing risk of pre-renal kidney injury, or concurrent use of several drugs resulting in potassium retention) was a common problem in community-dwelling PolSenior population. Method of kidney function assessment may influence the prevalence of potentially inappropriate use of such drugs. Assessment of kidney function with sMDRD-derived eGFR may result in overlooking of recommended dose adjustment formulated based on creatinine clearance.

## Abbreviations

ACEI: angiotensin-converting enzyme inhibitor
ARB: angiotensin II receptor blocker
CC: creatinine clearance
CKD: chronic kidney disease
eGFR: estimated glomerular filtration rate
NSAIDs: non-steroidal anti-inflammatory drugs
sMDRD: the short Modification of Diet in Renal Disease formula
